# The Development and Validation of the Health Behavior Motivation Scale

**DOI:** 10.3389/fpsyg.2021.706495

**Published:** 2021-09-03

**Authors:** Magdalena Poraj-Weder, Aneta Pasternak, Michał Szulawski

**Affiliations:** ^1^Institute of Psychology, The Maria Grzegorzewska University, Warsaw, Poland; ^2^Institute of Pedagogy and Psychology, Warsaw Management University, Warsaw, Poland

**Keywords:** motivation toward health behaviors, self-determination theory, motivational continuum, regulatory styles, questionnaire

## Abstract

The article presents the construction and validation process of the *Health Behavior Motivation Scale* (HBMS), which measures the motivation toward pro-health behaviors in population of healthy adults. The tool is conceptually based on Self-Determination Theory (SDT) and more precisely on one of its subtheories—Organismic Integration Theory (OIT). In the first stage of the construction, the linguistic validation with competent judges procedure allowed to eliminate the items which were not correctly formulated. Next, the psychometric properties of the HBMS were assessed in three studies. In Study 1 (*N* = 323, *M*_*age*_= 31), the factorial structure of the HBMS was assessed with CFA. Since the preliminary structure was rejected, in order to identify the dimensionality of the items, EFA and Horn's Parallel Analysis were performed. The results showed that the HBMS scale has 5–dimensional structure (intrinsic regulation, integrated and identified regulation, introjected regulation, external regulation and amotivation). In Study 2 (*N* = 342, *M*_*age*_= 33), the structure of the HBMS has been confirmed by conducting CFA analysis. Analyses preformed in this study provided good evidence for convergent and discriminant validity as well as the internal reliability of the HBMS subscales. Finally, in the LPA analysis two classes with distinct regulatory profiles have been extracted, which showed differences in the extend of health-related behaviors. In Study 3 (*N* = 60, *M*_*age*_= 30) the test–retest reliability of the HBMS was confirmed. The scale can be therefore successfully used in future basic and applied studies as it possesses robust psychometric properties.

## Introduction

**Figure 1:**
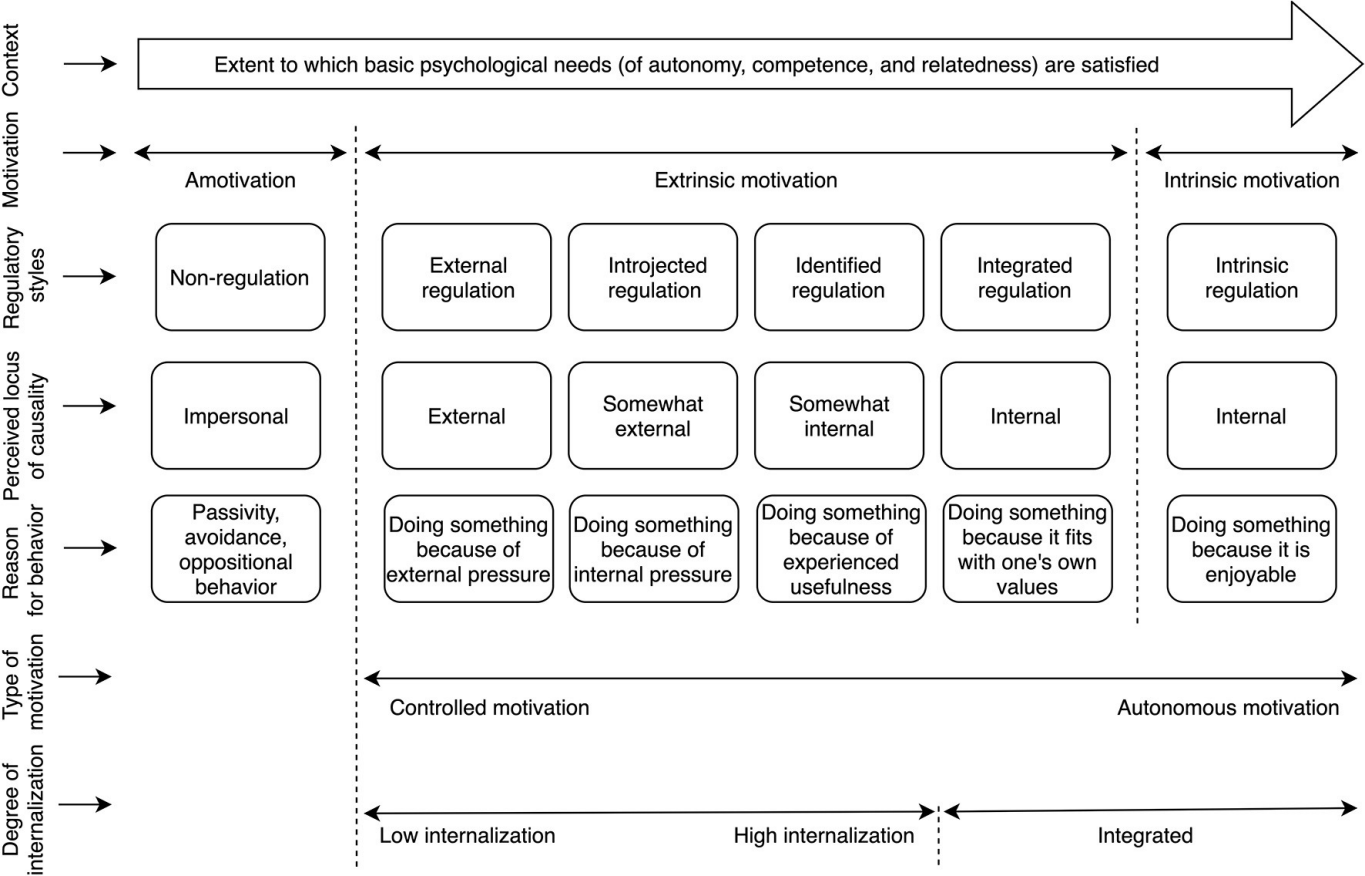
Schematic representation of Organismic Integration Theory. Source: Poraj-Weder et al. (2021).

For most people, health is probably one of the most important values in life. We wish others good health on numerous occasions, and if we were asked to say what we would wish for ourselves or our nearest, health again would probably be at the top of our list. Keeping good health is not only an important value, but also an important challenge in the modern world. Even though, people generally seem to value health, many strive to keep a balanced diet and do regular physical activity. As a consequence, a considerable number of the world population, including children and adolescents, is now overweight (Bray et al., 2017) with far-reaching consequences in terms of increased risk of chronic illness. Changing and keeping the healthy diet and exercising can have as powerful positive effect on health as the best medical interventions (Djoussé et al., 2009). For example, it is assessed that over 80% of type 2 diabetes and circulatory system diseases and at least 40% of cancer can be avoided through changes in behavior (World Health Organization (WHO), 2005). Despite the fact that in modern developed countries the good health behaviors depend mainly on us, as we decide of what kind of grocery shopping we do, and whether we spend our time on jogging or surfing through the Internet, changing our behaviors is not an easy task. One of the factors associated with the successes and failures of health behavior change is the quality of our motivation, the topic which is described and developed by the research in paradigm of Self-Determination Theory (SDT; Deci and Ryan, 2000; Vansteenkiste et al., 2009; Ryan and Deci, 2017). The aim of this research project was to create and validate a *Health Behavior Motivation Scale* (HBMS) which measures various forms of motivational regulation to undertake pro-health behaviors in the paradigm of SDT. According to SDT, and more precisely one of its subtheories—Organismic Integration Theory (OIT; Ryan and Deci, 2017)—there are six different types of motivational regulation, which vary in their antecedents, the degree of perceived autonomy, and effects on behavior. The motivational regulation styles could be represented in a form of continuum (see Figure 1), which represents the model of internalization and integration. The continuum starts with originally transmitted forms of external regulation and finishes with regulation fully integrated with one's values and personality, which at the same time, is full autonomous. Each type of regulation corresponds with the reason why one decides do behave in particular way. The first category, called amotivation is a state when one finds no meaning, value or eagerness to act in particular way mainly because they believe will not succeed in it (Ryan and Deci, 2017). An example of amotivation toward jogging is when one does not find it enjoyable, does not want to get fit, slim or healthy because one thinks that he or she will not succeed in jogging. Next, come the four types of extrinsic regulation, first of which is called external regulation. A behavior is regulated externally when a separable consequence—a reward, punishment or other outside pressure are its main reasons. This type of regulation is connected with external perceived locus of causality and perceiving the results of behavior as not dependent on one's actions. An adolescent who eats healthy food for additional pocket money from his parents or a person who exercises mainly to satisfy the expectations of his or hers personal trainer are examples of externally regulated pro-health behaviors.

Another type of extrinsic motivation is introjected regulation, which is a process of behavior regulation through internally demanding or pressuring force, a sense of I “must” or “should” do something. Introjected regulation is connected with feelings of anxiety and self-disparagement but can also be connected with self-pride and satisfaction. A situation when a person who feels that he or she has to go to the gym to exercise and feels wrong and ashamed if he or she didn't, may serve as an example of introjection. This type of motivation is also connected with external perceived locus of causality. Subsequent type of extrinsic motivation is identified regulation, which is associated with more autonomic behaviors and internal perceived locus of control. The person regulated through identification accepts values and standards connected with the behavior and perceives them as important. Still, the behavior is a means to some goal. An example may be eating healthier food in order to reduce weight or exercise in order to look prettier. The last, and the most autonomic form of extrinsic motivation according to SDT is integrated regulation, when the pro-health activity is not only personally important and valued (as it was the case with identified regulation), but it is also a natural consequence of one's identity and system of values. For example, when one perceives him/herself as a sports person then doing sports is a natural thing to do, it's an “idea for life” which belongs to the value system. This regulation, however, is still considered external as the behavior serves achieving some external goal—creating or confirming one's identity. The sixth, and last form of regulation distinguished is the intrinsic regulation, which is a separate category of the continuum. The intrinsically motivated pro-health behavior is fun and enjoyable for its own sake, the activity is treated as play, an opportunity to discover, and expand one's competencies and capacities. In other words, intrinsic motivation, in its purest sense, means “to play or explore an activity because it's itself is interesting” (Ryan and Deci, 2017, p. 123). Playing football for fun and pure enjoyment of the game might be an example of intrinsically motivated behavior. The OIT theory assumes that the more autonomously regulated the health behavior is, the greater effort, engagement, persistence, and stability that individual is likely to evidence in that behavior (Ryan and Deci, 2017). Consequently, in order to promote more stable and persistent change (or implementation) of particular type of behavior we need a proper understanding of the mechanisms which lie underneath.

### The Reasons Behind the HBMS Scale Construction

The reason behind the construction of the HBMS is the need of a measurement tool that considers motivation toward pro-health behaviors in a qualitative way and takes into consideration different styles of regulation. When developing the HBMS we referred to a category of health behaviors understood as “any activity undertaken by a person believing himself to be healthy, for the purpose of preventing disease or detecting it in an asymptomatic stage” (Kasl and Cobb, 1966, p. 531). Specifically, we concentrated on narrow category of pro-health behaviors (Conner and Norman, 1996; Sȩk, 2005) composed of personal routine daily health activities, named health practices (Harris and Guten, 1979). A conceptual framework of the HBMS was also based on the SDT (Ryan and Deci, 2000, 2017) and in particular on one of its subtheories—OIT (Deci and Ryan, 2000; Vansteenkiste et al., 2009; Ryan and Deci, 2017). The HBMS was thought as an operationalization of Ryan and Deci's concept of six different types of motivational regulation. These different forms of motivational regulation are conceptualized as lying along a continuum from non-autonomous to wholly autonomous forms of behavioral regulation. For the recent 30 years, this theory has been an important research area in the field of optimal human functioning in various social situations (Levesque et al., 2007; Pittman and Zeigler, 2007; Shah and Gardner, 2008; Vallerand et al., 2008; Chrupała-Pniak and Grabowski, 2016). Nevertheless, undertaking pro-health behaviors is still an insufficiently explored issue in the context of human motivation. Poland also lacks diagnostic tools that would allow for the measurement of this theoretical construct in a health-related context. The only tools, which are currently available, measure motivation toward different then health activities, such as work (Bańka, 2005; Wojdyło and Retowski, 2012; Chrupała-Pniak and Grabowski, 2016) or study (Gózdz, 2015). The construction of the HBMS seems to fill this gap. Currently, the only questionnaire that allows to diagnose the qualitative facets of motivation (according to SDT) is the *Treatment Self-Regulation Questionnaire* (TSRQ) (Ryan and Connell, 1989; Levesque et al., 2007). The TSRQ is a tool that measures pro-health behaviors in four different contexts—quitting smoking, reducing of alcohol use, changing eating habits, and changing physical exercise patterns (Levesque et al., 2007). The tool, although widely used, has some limitations. The questionnaire is limited to only one of the four contexts of pro-health behavior and may selectively diagnose only three of the six regulative styles (amotivation, external regulation and introjected regulation) and one broader motivational composite which is autonomous motivation. Although, according to Vallerand (1997) and Vallerand and Ratelle (2002) autonomous motivation includes three regulatory styles (identified, integrated and intrinsic regulation), the TSRQ lacks items operationalizing the third one (intrinsic regulation). The tool has also varied number of items for each of the subscales and in two subscales, the number of items is too low: 3 items for amotivation subscale and 2 items for introjected regulation subscale, respectively. According to the methodological literature (Zawadzki, 2006; Brzeziński, 2007) low number of items in a scale may reduce the reliability of the tool.

The project of creating and validating of the HBMS was designed to address the limitations of the TSRQ. When developing the HBMS, we aimed to make it a valuable alternative to the TSRQ. The new questionnaire was supposed to capture all the six regulatory styles described within motivational continuum in a more generic health context. Such a scale could be used for both scientific and practical purposes, providing healthcare professionals with helpful tool for planning health promotion interventions with their patients or clients.

## Research Problem and Hypotheses

The main goal of this study was to construct and validate the *Health Behavior Motivation Scale* (HBMS) for assessing the motivation toward pro-health behaviors in population of healthy adults. For this purpose, three studies were conducted. Study 1 served to identify the factor structure of the HBMS. In Study 2 we aimed to verify the factor structure developed in Study 1 and assess the HBMS psychometric properties (discriminant and convergent validity and the internal reliability). Study 2 also served to corroborate if in the given population it is possible to sub-groups, that would differ significantly in terms of their regulatory profile. We also wanted to determine whether there is a relationship between the extracted HBMS profiles and health-related behaviors. Finally, in Study 3 we aimed to assess the test–retest reliability of the HBMS.

In terms of structural validity, we assumed that the factor structure of the HBMS would correspond with the OIT taxonomy of regulatory styles (Ryan and Deci, 2000, 2017) (H1). Based on the Ryan and Connell (1989) and Deci and Ryan (2000) we expected that latent variables representing individual components of the regulatory styles will be correlated with each other. We also anticipated that dimensions, representing the regulatory styles, that are closer together along the continuum will be more highly correlated than those theorized to be more distant (Ryan and Connell, 1989) (H2).

In terms of convergent and discriminant validity, four hypotheses were formulated: we expected that the HBMS subscales would be correlated with the selected subscales of the *Aspiration Index* (Kasser and Ryan, 1993, 1996), the *Promotion and Prevention Self-Regulation Scale* (Kolańczyk et al., 2013), the *Sociocultural Attitudes Toward Appearance* (Schaefer et al., 2015) and the *Multidimensional Body-Self Relations Questionnaire* (Cash, 2000). Specifically, we expected that intrinsic aspirations and health aspirations will be positively correlated with autonomous forms of motivational regulation (e.g., intrinsic or identified and integrated regulation), and negatively correlated with controlled forms of motivational regulation (e.g., external regulation) (Kasser and Ryan, 1996; Piko and Keresztes, 2006) (H3). With respect to the regulatory focus (Higgins, 1998), we expected that promotion focus will be associated with autonomous forms of motivational regulation and negatively with controlled forms of motivational regulation and amotivation. Prevention focus, on the other hand, should be related to controlled forms of regulation, especially introjected regulation. Motivation strength was expected to be negatively related to amotivation, and positively related to autonomous forms of motivational regulation (Deci and Ryan, 2000) (H4). Based on SDT theory and literature reports (De Charms, 1968; Nicholls, 1984; Williams et al., 1996; Deci and Ryan, 2000; Chrupała-Pniak and Grabowski, 2016; Raposo et al., 2020) we expected that different types of ideal appearance pressures exerted by family, media and peers will be positively correlated with controlled forms of motivational regulation (external regulation, introjected) and amotivation (H5) Based on literature reports (Williams et al., 1998; Levesque et al., 2007; Vallerand et al., 2008; Juczyński, 2012), we also expected that the positive evaluation of fitness and health, as well as orientation to these aspects of the physical self in the MBRSQ will be positively correlated with the autonomous forms of motivational regulation (intrinsic, integrated and identified regulation) and negatively with external regulation and amotivation. Moreover, positive associations were expected between health and fitness orientation and introjected regulation (Williams et al., 1998; Levesque et al., 2007) (H6). Finally, we expected that sub-groups extracted in the LPA with lower levels of autonomous forms of motivational regulation would declare lower levels of healthy eating habits, preventive health behaviors and health practices as well as less positive mental attitude toward health measured by the *Health Behavior Inventory* (Juczyński, 2012) when compared to the sub-groups with higher levels of autonomous forms of motivational regulation (H7).

### Construction of the HBMS Questionnaire

The HBMS scale development was based on recommended, theoretical procedure of diagnostic measures construction (Deci and Ryan, 1985; Magnusson, 1991; Deci et al., 1994; Ryan and Deci, 2000; Zawadzki, 2006; Brzeziński, 2007; Hornowska, 2007). Seventy test items included in six scales (which constituted the operationalization of six regulatory styles) were generated based on analyzing the conceptual assumptions of SDT theory (Deci and Ryan, 1985; Deci et al., 1994; Ryan and Deci, 2000). This stage of the tool's construction process was carried out with the participation of two psychologists specializing in issues of health psychology and research on motivation based on SDT.

In the next step, the generated items were subjected to linguistic and content analyses (Hornowska, 2007). The linguistic assessment was carried out by a team of three psychologists and two Polish philologists. All the items were checked in terms of their vocabularies' degree of comprehensibility, grammatical correctness as well as length and complexity (Hornowska, 2007). Nine items that were assessed as incomprehensible or too lengthy were eliminated. The version of the HBMS, reduced to 61 items, was subjected to further content analysis aimed at determining its compliance with the theoretical construct. The analysis was conducted by a group of competent judges, consisting of three psychologists specializing in research on motivation in terms of SDT and having psychological practice in motivating people in the professional environment. The team included a certified sports psychologist of the Polish Psychological Association, a member of the Polish Olympic Committee and the Central Center of Sports Medicine. Each judge was given the definition of the six regulatory styles (presented in the theoretical introduction to the manuscript). The judges were then asked to allocate each of the 61 initial test items to one of the six regulatory styles. Kendall's *W* coefficient was used as a measure of the judge's score compliance. On the basis of the feedback provided by the judges, the number of items was reduced to 52. Ambiguous items, difficult to assign to one specific dimension were eliminated. The coefficient's value, *W* = 0.94, $\chi_{\left(51\right)}^{2}$ = 143,72, *p* < 0.001, proved a high degree of agreement between the judges' assessment and did not provide grounds for further reducing the scale. Consequently, 52 statements were found in the HBMS' first version. At the end of this stage, the questionnaire's administrative issues were established. These included authoring an author's note and its name, clarifying the instructions contained in it, and determining the format of responses and the order of items in the questionnaire. All items contained in HBMS were given the nature of closed statements with the possibility of providing one of five categories of answers: 0–*this statement does not suit me at all*, 1–*this statement suits me very poorly*, 2–*this statement suits me poorly, 3–this statement suits me on average*, 4–*this statement suits me well*, 5–*this statement suits me very well, 6 –undecided*. The last category of answers (6–*undecided*) at the analysis stage was treated as missing data. Such prepared scale was used in three studies in order to assess its psychometric properties: validity and reliability.

## Study 1

The aim of Study 1 was to investigate structural validity and internal consistency of the HBMS.

### Procedure and Participants

The research project was conducted on 734 healthy adults (without any chronic illnesses) recruited from a non-clinical population. Three samples were tested, Study 1 (*N* = 332), Study 2 (*N* = 342) and Study 3 (*N* = 60). The sample sizes in Studies 2 and 3 were determined in accordance with the recommendations formulated by Mundfrom et al. (2009) regarding the minimum required to perform factor analyses.

A purposive sampling approach was used in the study (Brzeziński, 2006). Inclusion criteria encompassed being 18 years of age or older, no history or presence of chronic medical. Available people—volunteers meeting the above criteria—participated in the study. There was no remuneration for participation.

Data was collected from November 2020 to February 2021. The participants of the study were recruited among university students and employees of various workplaces, enterprises and companies throughout Poland. All three studies were conducted individually, using the paper-and-pencil method, in the presence of a researcher. The project was conducted in accordance with the recommendations of the Code of Ethics for the Psychologist of the Polish Psychological Society (Polish Psychological Association [PTP], 1992). The protocol of this study was accepted by the ethics committee of the Maria Grzegorzewska University. All participants provided informed consent.

Information on the sample structure is presented in Table 1. Apart from the sociodemographic variables, the description of the sample (in Studies 1, 2, and 3) also includes variables describing the subjective assessment of health status and the degree of implementation of pro-health behaviors and their regularity.

**Table 1:** Summary of the characteristics of the tested samples, Study 1–3.

	**Study 1**	**Study 2**	**Study 3**
Sample size	332	342	60
Gender			
Female	206 (62%)	179 (52.3%)	24 (40%)
Male	126 (38%)	163 (47.7%)	36 (60%)
Age in years (*M* ±*SD*)	31.49 ± 12,32	33.29 ± 10.07	29.80 ± 5.01
Education			
Elementary	21 (6.3%)	10 (2.9%)	4 (6.7%)
Vocational	21 (6.3%)	23 (6.7%)	1 (1.7%)
Secondary	158 (47.6%)	135 (39.5%)	22 (36.7)
Higher education	132 (39.8%)	174 (50.9%)	33 (55%)
Place of residence			
Village or small town up to 20 thousand residents	93 (28%)	53 (15.5%)	4 (6.7%)
City 21–100 thousand residents	44 (13.3%)	41 (12%)	5 (8.3%)
City 101–500 thousand residents	35 (10.5%)	45 (13.2%)	6 (10%)
City over 500 thousand residents	160 (48.2%)	203 (59.4%)	45 (75%)

### Measures

Participants completed the HBMS questionnaire and the sociodemographic survey, containing questions about the health assessment and implementation of the pro-health behaviors.

### Statistical Analyses

**Figure 2:**
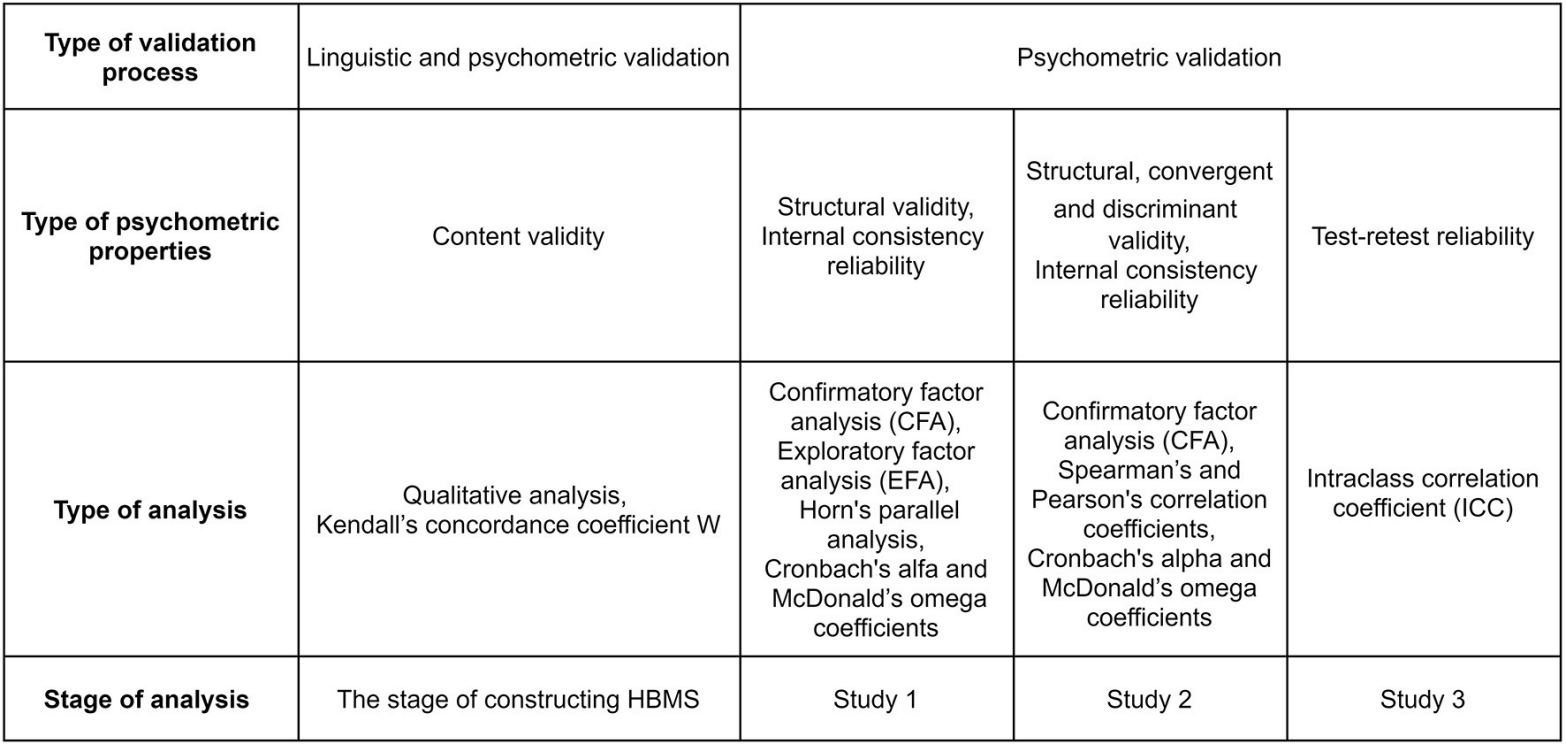
HBMS validation procedure.

The analyzes used in Study 1 are presented in Figure 2. Statistical analyses were performed using IBM SPSS Statistics 26 software (IBM Corporation, 2019) and AMOS 26.0 software (Arbuckle, 2019).

#### Structural Validity and Internal Reliability

In order to verify the factorial structure of the 52-item version of the HBMS confirmatory factor analysis (CFA) based on the likelihood method was conducted. To determine the adequacy of the model fit, two criteria were used: RMSEA (*root mean of square error of approximation*) and CFI (*comparative fit index*). The first criterium is a measure of model-to-data mismatch (Byrne, 2010). The second one is used to assess the quality of the model's fit by comparing it with the variance-covariance matrix (Byrne, 2010). In publications devoted to structural modeling, it is assumed that the RMSEA value should be as close to zero as possible (Byrne, 2010), whereas CFI index should have values above 0.95 (Hu and Bentler, 1999, as cited in Byrne, 2010). However, the preliminary structure did not yield adequate fit. The values of fit indices were equal to *CFI* = 0.84, *RMSEA* = 0.08. Therefore, the preliminary structure was rejected, and exploratory factor analysis (EFA) was performed. The values of factor loadings acquired in the CFA are presented in Supplementary Table 1A.

In the next step, in order to verify identify the dimensionality of the items, the EFA was performed, using a Principal Component Analysis (PCA) with Oblimin rotation and Kaiser normalization. This rotation was selected because of the assumed possibility of factor correlations (Tabachnick and Fidel, 2007). Components were identified based on sedimentation (scree plot) graphs (Izquierdo et al., 2014).

**Figure 3:**
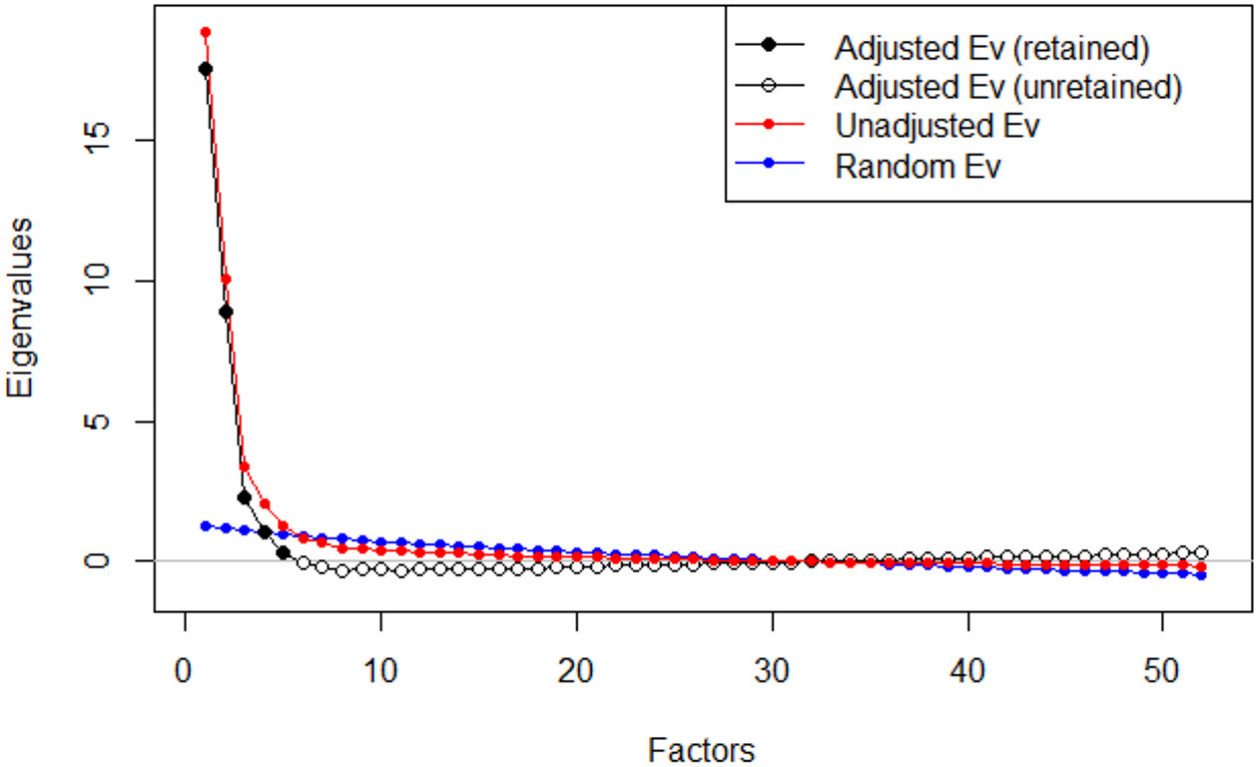
Plot for Horn's Parallel Analysis.

The measures of the sample selection's adequacy were satisfactory. Bartlett's Test of Sphericity was significant (χ^2^ = 12,381.40, *df* = 1,326, *p* < 0.001), and the KMO measure of sampling adequacy was 0.94. The obtained results indicate a good fit of the model to the data and constitute the basis for the use of EFA in assessing relationships between observable variables (Field, 2005). The analysis showed six main components with an eigenvalue above one, explaining a total of 72.55% of the variance. However, due to the shape of the scree plot, clearly showing five components, Horn's parallel analysis was executed. “The method compares the eigenvalues generated from the data matrix to the eigenvalues generated from a Monte-Carlo simulated matrix created from random data of the same size” (Allen, 2017, p. 518). In Horn's Parallel Analysis, the eigenvalues of the EFA should be higher than those obtained from the parallel analysis (Horn, 1965). The eigenvalue of the fifth factor in the actual data (EFA) is 1.49 and it's <1.61 in the simulative data of Parallel Analysis. In that case, the factor five should be considered as the point at which Parallel Analysis introduces a decision about the number of factors. The results of our analyzes are presented in Table 2 and illustrated in the Figure 3.

**Table 2:** Eigenvalues of EFA and random data - parallel analysis using the Horn's method, Study 1.

**Component**	**Eigenvalues** **(EFA)**	**Eigenvalues generated from** **random data (Horn's parallel analysis)**
1	19.06	1.86
2	10.28	1.78
3	3.62	1.72
4	2.31	1.66
5	1.49	1.61
6	1.10	1.56

As a result of the conducted analyzes, the 5-factor solution was finally adopted. The results of Bartlett's Test of Sphericity and the KMO measure were: χ^2^ = 12,381.40, *df* = 1,326, *p* < 0.001; 0.94, respectively. After the rotation, the first factor explained 36.67% of the variance, and the next ones were: 19.51, 6.95, 4.46, and 2.84%. The five factors derived from the EFA account for 70.44% of the common variance. In the next step, within each dimension, six items with the highest factor loadings were selected. Thus, the final version, consisting of 30 test items was obtained.

In the next analysis, the internal structure of the final version of the scale, composed of 30 statements, was examined. Again, the five-component EFA was used, performed using the principal component method, with Oblimin rotation and Kaiser normalization. Bartlett's Test of Sphericity was significant (χ^2^ = 6,765.51, *df* = 435, *p* < 0.001) and the sampling adequacy measure was high (KMO = 0.92) (Field, 2005). The total variance explained by the model was 75.72% (Factor 1: 31.02%, Factor 2: 25.08%, Factor 3: 9.39%, Factor 4: 6.26%, and Factor 5: 3.96%). Table 3 lists the factor loadings of the five factors extracted in the final solution.

**Table 3:** Factor loadings in EFA for 30-item Health Behavior Motivation Scale (HBMS), Study 1.

**HBMS dimensions**	**Factor 1**	**Factor 1**	**Factor 3**	**Factor 4**	**Factor 5**
**Intrinsic regulation**					
**α = 0.95, 95% Cl (0.93; 0.96); ω = 0.95, 95% Cl (0.93; 0.96)**					
49_Because it makes me happy	0.91				
45_ Because it gives me vitality	0.90				
40_ Because it gives me vigor	0.89				
54_Because it gives me pleasure	0.84				
59_Because it's a lot of fun	0.81				
27_Because it drives me to act	0.77				
**Identified and integrated regulation**					
**α = 0.90, 95% Cl (0.88; 0.92); ω = 0.90, 95% Cl (0.88; 0.92)**					
9_Because I treat it as an important and ongoing task to undertake					0.86^*^
8_Because it's my life plan					0.83^*^
23_Because it is my current life choice					0.75^*^
15_Because it is an essential part of my life					0.73^*^
16_Because the “here and now” is important for me					0.72^*^
36_Because it is congruent with my currently set life goals					0.70^*^
**Introjected regulation**					
**α = 0.91, 95% Cl (0.90; 0.93); ω = 0.92, 95% Cl (0.90; 0.93)**					
37_Because whenever I neglect my health I feel guilty			0.91^*^		
46_Because I feel remorse when I neglect my health			0.91^*^		
42_Because I feel remorse when my health becomes less of a priority			0.84^*^		
30_Because I feel guilty when I stop taking care of my health			0.75^*^		
10_Because I feel remorse if I don't take care of my health			0.74^*^		
24_Because if I don't take care of my health I feel like I'm acting wrong			0.58^*^		
**External regulation**					
**α = 0.93, 95% Cl (0.91; 0.94); ω = 0.93, 95% Cl (0.91; 0.94)**					
5_Because others expect me to take care of my health				0.86^*^	
31_Because I want to meet others' expectations				0.85^*^	
25_Because I want to make others happy				0.82^*^	
18_Because I don't want to disappoint people around me				0.79^*^	
20_Because I feel pressure from my social environment				0.78^*^	
61_ Because I don't want others to nitpick my actions				0.77^*^	
**Amotivation**					
**α = 0.93, 95% Cl (0.91; 0.96); ω = 0.93, 95% Cl (0.91; 0.95)**					
26_I don't do it because it causes me to feel lost		0.89			
32_ I don't do it because a feeling of helplessness arises in me		0.87			
39_I don't do it because I could fail again		0.86			
58_I don't do it because I feel like I can't		0.84			
19_I don't do it because it's beyond me		0.84			
48_ I don't do it because I don't have energy		0.80			

* *Reversed scale*.

Next, Cronbach's alpha (α) and McDonald's omega (ω) were used to assess the internal consistency of each factor. The values of Cronbach's alpha ranged 0.91–0.94, and McDonald's omega 0.83–0.94. The results indicate high reliability of the five dimensions of the HBMS (Table 3).

### Discussion

Based on our analyses, we can conclude that the HBMS is a structurally valid and reliable tool.

Both, CFA and EFA with Horn's Parallel Analysis, resulted in emergence of five-factor model corresponding with the motivational continuum by Ryan and Deci (2000, 2017). The following factors were extracted: intrinsic regulation, integrated and identified regulation, introjected regulation, external regulation and amotivation. Although, the model corresponds to the theoretical OIT taxonomy of regulatory styles, the number of factors is reduced, therefore H1 was only partially confirmed. In our analysis, identified regulation and integrated regulation, which are originally distinct, constitute one dimension.

Similar difficulties encountered the authors of the TSRQ (Levesque et al., 2007). In their validation studies (Levesque et al., 2007) intrinsic, identified and integrated regulation formed one dimension (autonomous regulation). Explanations that help to understand these results can be found in the characteristics of the integration dimension and the difficulties with its operationalization, as pointed out by other researchers (Vallerand, 1997; Meyer and Gagne, 2008; Chrupała-Pniak and Grabowski, 2016). The combining the dimension of identified and integrated regulation into one general composite has its justification in the subject's literature (Deci and Ryan, 2000, 2008). Both dimensions still belong to external motivation, but already autonomously regulated. The difference between them essentially comes down to the fact that in the case of identified regulation, individuals identify themselves with a set of values and meanings, accepting them as their own, while in the case of integrated regulation a given value or meaning falls within the scope of one's identity (Deci and Ryan, 2000).

## Study 2

The aim of Study 2 was to verify the factor structure developed during EFA and Horn's Parallel Analys is in Study 1 and assess the HBMS psychometric properties (discriminant and convergent validity and the internal reliability).

### Procedure and Participants

The sample in Study 2 was comprised of 342 healthy adults. The detailed description of the sample structure is presented in Table 1.

### Measures

Participants completed a set of six questionnaires and the demographic survey.

#### Aspiration Index

Aspiration Index (AI-23) is grounded in SDT and measures the content of life goals. It was created by Kasser and Ryan (1993, 1996) and adapted to Polish by Górnik-Durose et al. (2018). The AI-23 contains 23 items and seven specific subscales, which make up three categories of goals: intrinsic, extrinsic and self-transcendent. Respondents assess the importance of 23 goals on a five-point scale, ranging from 1–*not at all important* to 5–*very important*. A higher total score on each scale indicates a higher importance of certain goal category. AI-23 has satisfactory psychometric parameters (Górnik-Durose et al., 2018). In the present study, the reliability measured by Cronbach's α coefficient varied between 0.69 and 0.82. In our analyses, we referred to the intrinsic aspirations of meaningful relationships, personal growth, and community contributions, as well as aspiration of good health.

#### Promotion and Prevention Self-Regulation Scale

Promotion and Prevention Self-Regulation Scale (PPSS) (Kolańczyk et al., 2013) is a measure of promotion and prevention regulatory focus. The tool is based on Higgins (1987) theory, as well as on the results of the studies on emotionality of people differing in dispositional regulatory focus. It contains 27 items within three subscales: promotion, prevention and strength of motivation. Respondents assess each item on five-point scale, ranging from 1–*strongly disagree* to 5–*strongly agree*. The tool has satisfactory psychometric parameters (Kolańczyk et al., 2013). In our study Cronbach's α coefficient ranged from 0.76 (strength of motivation) to 0.82 (promotion focus).

#### Multidimensional Body-Self Relations Questionnaire

Multidimensional Body-Self Relations Questionnaire (MBSRQ) (Cash, 2000) is a well-validated measure of body-image attitudes. It was created by Cash (2000) and adapted by Schier, Rzeszutek, Topór, Matkowska and Pasternak (Pasternak, 2018). The tool contains 69 items and ten subscales, describing different aspects of the attitude to the body image including evaluative, cognitive and behavioral components. In the present study four subscales were used: the fitness and health evaluation subscale and the fitness and health orientation subscale. High scorers consider themselves as physically fit, healthy and try to lead a healthy lifestyle. Respondents assess each item on five-point scale, ranging from 1–*definitely disagree* to 5–*definitely agree*. The MBRSQ has satisfactory psychometric parameters (Cash, 2000). In our study, Cronbach's α coefficient for these subscales varied between 0.80 and 0.91.

#### Sociocultural Attitudes Toward Appearance

Sociocultural Attitudes Toward Appearance (SATAQ-4) was used to measure internalization of appearance ideals as well as perceived sociocultural pressures related to the appearance including three different aspects (family, peers and media) (Schaefer et al., 2015). In our study we used Polish adaptation. The tool contains 22 items within five subscales: internalization: thinness, internalization: muscularity, pressures: family, pressures: media and pressures: peers. In the present study only subscales regarding to pressures were used. Respondents assess each item on a five-point scale, ranging from 1–definitely disagree to 5–definitely agree. A higher total score indicates a higher pressure perceived to reach such ideals. The SATAQ-4 has satisfactory psychometric parameters (Schaefer et al., 2015). In our analyses, Cronbach's α coefficient for these subscales ranged between 0.92 and 0.96.

#### Health Behavior Inventory

Health Behavior Inventory (IBH) is a (Juczyński, 2012) measure of health-related behaviors. It contains 24 statements within four subscales: healthy eating habits, preventive health behaviors, health practices and positive mental attitude toward health, connected with avoiding strong emotions, tensions and stresses. Respondents assess each item on five-point scale, ranging from 1–almost never to 5–almost always. A higher total score indicates a higher frequency in implementing health-related behaviors. The inventory has good statistical parameters (Juczyński, 2012). In our study, Cronbach's α coefficient ranged between 0.62 and 0.82.

### Statistical Analyses

The data analysis in Study 2 consisted of two consecutive steps.

The first series of analysis (presented in Figure 2) served to assess the validity of the HBMS. Further analysis aimed to define characteristic classes that differed regarding their profile of regulatory type. This approach is an application of a person-centered perspective, which in contrast to variable-centered approach, takes into account the heterogenity of participants within the studied variables. We extracted two classes that differed regarding their regulatory profile and examined the differences between them in terms of health-related behaviors. Statistical analyses were performed using IBM SPSS Statistics 26 software (IBM Corporation, 2019) and AMOS 26.0 software (Arbuckle, 2019). LPA was carried out in R Statistics software with the use of tidyLPA package.

### Results

#### Structural Validity and Internal Reliability

When constructing the model for analysis, we assumed, based on the Ryan and Connell (1989) and Deci and Ryan (2000) that latent variables representing individual components of the regulatory styles will be correlated with each other (H2). The parameter values were estimated using the maximum likelihood method. Again, to determine the adequacy of the model fit, two criteria were used: *RMSEA* and *CFI*. The measures of *RMSEA* = 0.053 and *CFI* = 0.955 reached values that yield a good fit of the data to the HBMS model. All items constituting HBMS dimensions had significant factor loadings, which confirms the model developed in Study 1.

In the next step, we analyzed the internal consistency of the HBMS scales in this sample. All of them achieved a satisfactory level of reliability measured by Cronbach's α and ω coefficients. We decided to provide ω coefficients as a more adequate measure of reliability, still keeping Cronbach's α as most commonly used index of reliability (Hayes and Coutts, 2020). The results of both analyses are displayed in Table 4.

**Table 4:** Factor loadings in CFA for 30-item health behavior motivation scale (HBMS), Study 2.

**HBMS dimensions**		***f***	***p***
Intrinsic regulation			
α = 0.95, ω = 0.95	Item 27	0.85	0.001
	Item 40	0.89	0.001
	Item 45	0.87	0.001
	Item 59	0.87	0.001
	Item 54	0.87	0.001
	Item 49	0.89	0.001
Integrated and identified regulation			
α = 0.90, ω = 0.90	Item 15	0.84	0.001
	Item 36	0.75	0.001
	Item 23	0.83	0.001
	Item 8	0.76	0.001
	Item 16	0.69	0.001
	Item 9	0.73	0.001
Introjected regulation			
α = 0.88, ω = 0.88	Item 24	0.69	0.001
	Item 30	0.63	0.001
	Item 10	0.66	0.001
	Item 42	0.85	0.001
	Item 46	0.80	0.001
	Item 37	0.81	0.001
External regulation			
α = 0.93, ω = 0.93	Item 20	0.85	0.001
	Item 61	0.83	0.001
	Item 18	0.79	0.001
	Item 31	0.91	0.001
	Item 25	0.92	0.001
	Item 5	0.67	0.001
Amotivation			
α = 0.93, ω = 0.93	Item 48	0.81	0.001
	Item 58	0.82	0.001
	Item 39	0.85	0.001
	Item 32	0.87	0.001
	Item 19	0.86	0.001
	Item 26	0.82	0.001

#### Intercorrelations Between HBMS Dimensions

In the next step, the intercorrelations between the HBMS dimensions were assessed. As expected (H2), scales associated with higher degree of perceived autonomy (intrinsic regulation, identified and integrated regulation) correlated positively with each other and negatively with External regulation and Amotivation. The maximum value of Pearson's r correlation coefficient was obtained for the Intrinsic regulation and Identified and integrated regulation. Also scales associated with lower degree of perceived autonomy (external regulation and amotivation and external regulation and introjected regulation) were positively and highly intercorrelated. Positive and moderate value of correlation coefficient was found for external and introjected regulation (both associated with an external locus of causality). External regulation and identified and integrated regulation did not correlate with each other. The obtained results are displayed in Table 5. Based on modification index values, with the threshold value of 4, the model included correlations between items: 24 and 10 (constituting the *Introjected regulation* subscale), as well as correlations between items 20 and 25 (constituting the *External regulation* subscale).

**Table 5:** Intercorrelations of the health behavior motivation scale (HBMS) dimensions, Study 2.

**HBMS dimensions and items**	**HBMS dimensions and items**	***r***	***p***
Intrinsic regulation	Integrated and identified regulation	0.86^**^	0.001
Intrinsic regulation	Introjected regulation	0.42^**^	0.001
Intrinsic regulation	External regulation	−0.15^**^	0.001
Intrinsic regulation	Amotivation	−0.27^**^	0.001
Integrated and identified regulation	Introjected regulation	0.47^**^	0.001
Integrated and identified regulation	Amotivation	−0.22^**^	0.001
Introjected regulation	External regulation	0.39^**^	0.001
Introjected regulation	Amotivation	0.15^**^	0.001
External regulation	Amotivation	0.68^**^	0.001
Item no. 24	Item no. 10	0.37^**^	0.001
Item no. 20	Item no. 25	−0.48^**^	0.001

*p < 0.01,

** *p < 0.001*.

#### Differences in the HBMS Dimensions for Sociodemographic Variables

Next a comparison of the intergroup differences in the HBMS dimensions in terms of sociodemographic variables (including gender, age, education and place of residence) was performed. According to the Student's *t*-test values for the independent samples, there was a statistical difference between men and women regarding introjected regulation, *t*_(338)_ = 2.27, *p* < 0.05. Within this dimension, women scored significantly higher (*M* = 17.96, *SD* = 7.71) than men (*M* = 15.98, *SD* = 8.35). We also examined the differences in the HBMS dimensions based on age, the education level and the size of the place of residence. Our analysis showed that there is a statistical difference in types of motivational regulation conditioned to the size of the place of residence, *t*_(269, 78)_ = 2.61, *p* < 0.05). People who live in small and middle cities have higher level of amotivation (*M* = 8.54, *SD* = 8.30) compared to the big city dwellers (*M* = 6.26, *SD* = 7.31). We did not find any differences in the HBMS dimensions conditioned to age or level of education. The results of our analyzes are included in the (Supplementary Tables 2A–5A).

#### Convergent and Discriminant Validity

In the next step, the construct validity of the HBMS was verified (H3–H6). First, correlations with the AI-23 (Kasser and Ryan, 1993, 1996) and the SSPP (Kolańczyk et al., 2013) were assessed (H3–H4). The obtained results are presented in Table 6.

**Table 6:** Pearson's and Spearman's correlation coefficients between the health behavior motivation scale (HBMS) subscales and different dimensions of AI23 and SSPP, Study 2.

**HBMS subscales**	**AI23 subscales**		**SSPP subscales**		
	**Health aspiration**	**Intrinsic aspiration**	**Promotion orientation**	**Prevention orientation**	**Motivation strength**
Intrinsic regulation	0.493^**^	0.323^**^	0.336^**^	0.108^*^	0.227^**^
Identified and integrated regulation	0.427^**^	0.187^**^	0.325^**^	0.151^**^	0.177^**^
Introjected regulation	0.292^**^	0.168^**^	0.134^**^	0.272^**^	−0.039
External regulation	−0.048	−0.146^**^	−0.079	0.214^**^	−0.175^**^
Amotivation	−0.174^**^	−0.245^**^	−0.218^**^	0.035	−0.295^**^

** *p < 0.01;*

* *p < 0.05 (one tailed); intrinsic aspiration coefficients are Rho-Spearman, all the other coefficients are r-Pearson*.

The correlation matrix between the HBMS and the AI-23 subscales showed positive correlations between health (moderate) and intrinsic aspirations (weak and moderate correlations) and intrinsic, identified and integrated regulation (autonomous forms of regulation) (H3). The correlations were stronger for health aspiration index than for the intrinsic aspiration index. Moreover, both health aspiration and intrinsic aspiration indices showed moderate positive correlations with introjected regulation. Intrinsic aspiration index, correlated negatively with external regulation and amotivation, whereas health specific aspiration did not correlate with external regulation, but correlated negatively with amotivation. These results confirm hypothesis H3.

The HBMS scales also correlated with the SSPP subscales (H4). When it comes to general motivation strength and promotion focus scales, they were both positively weakly associated with intrinsic regulation and identified and integrated regulation (autonomous forms of regulation). Moreover, motivation strength showed weak negative correlations with external regulation and amotivation and promotion focus was positively weakly associated with introjected regulation and negatively with amotivation. The prevention focus, on the other hand, showed moderate positive correlations with introjected and external regulations and weak positive correlations with intrinsic, integrated and identified regulations. These results confirm hypothesis H4.

Next the correlations between the HBMS and various dimensions of body image measured by the SATAQ-4 (Schaefer et al., 2015) and the MBRSQ (Cash, 2000) were assessed (H5–H6). The results of this analysis are presented in Table 7.

**Table 7:** Pearson's correlation coefficients between the health behavior motivation scale (HBMS) subscales and different dimension of SATAQ-4 and MBRSQ, Study 2.

**HBMS subscales**	**SATAQ-4 subscales**		**MBRSQ subscale**		
	**Pressures:** **family**	**Pressures:** **media**	**Pressures:** **peers**	**Fitness and** **health orientation**	**Fitness and** **health evaluation**
Intrinsic regulation	−0.087	−0.088	−0.016	0.592^**^	0.345^**^
Identified and integrated regulation	−0.009	0.057	0.036	0.598^**^	0.279^**^
Introjected regulation	0.059	0.219^**^	0.117^*^	0.194^**^	−0.065
External regulation	0.368^**^	0.422^**^	0.416^**^	−0.166^**^	−0.289^**^
Amotivation	0.294^**^	0.267^**^	0.323^**^	−0.314^**^	−0.343^**^

** *p < 0.01;*

* *p < 0.05 (one tailed)*.

The analysis of correlation matrix between the HBMS and the SATAQ-4 scales (Table 7) revealed the existence of weak and moderate, positive correlations between the different types of ideal appearance pressures exerted by family, media and peers and controlled forms of motivational regulation (introjected and external regulation) and amotivation. The strongest correlations of moderate size were obtained for external regulation. The only dimension, which was not significantly associated with introjected regulation was family pressure. In addition, no statistically significant correlations were observed between the SATAQ-4 dimensions and autonomous forms of regulation - intrinsic, identified and integrated regulation. The correlation pattern is as expected and confirms H5.

Moreover, an analysis of the obtained results showed that the positive evaluation of fitness and health and orientation to these aspects of the physical self in the MBRSQ are moderately positively correlated with the autonomous forms of motivational regulation (intrinsic, integrated and identified regulation) and weakly negatively correlated with external regulation and amotivation. Additionally, introjected regulation was weakly, positively correlated with health and fitness orientation. The strongest correlations were obtained for intrinsic regulation and amotivation. No significant relations were found only between the introjected regulation and fitness and health evaluation. The obtained results confirmed H6 (Valentine and Cooper, 2003).

#### Profile Analysis

**Figure 4:**
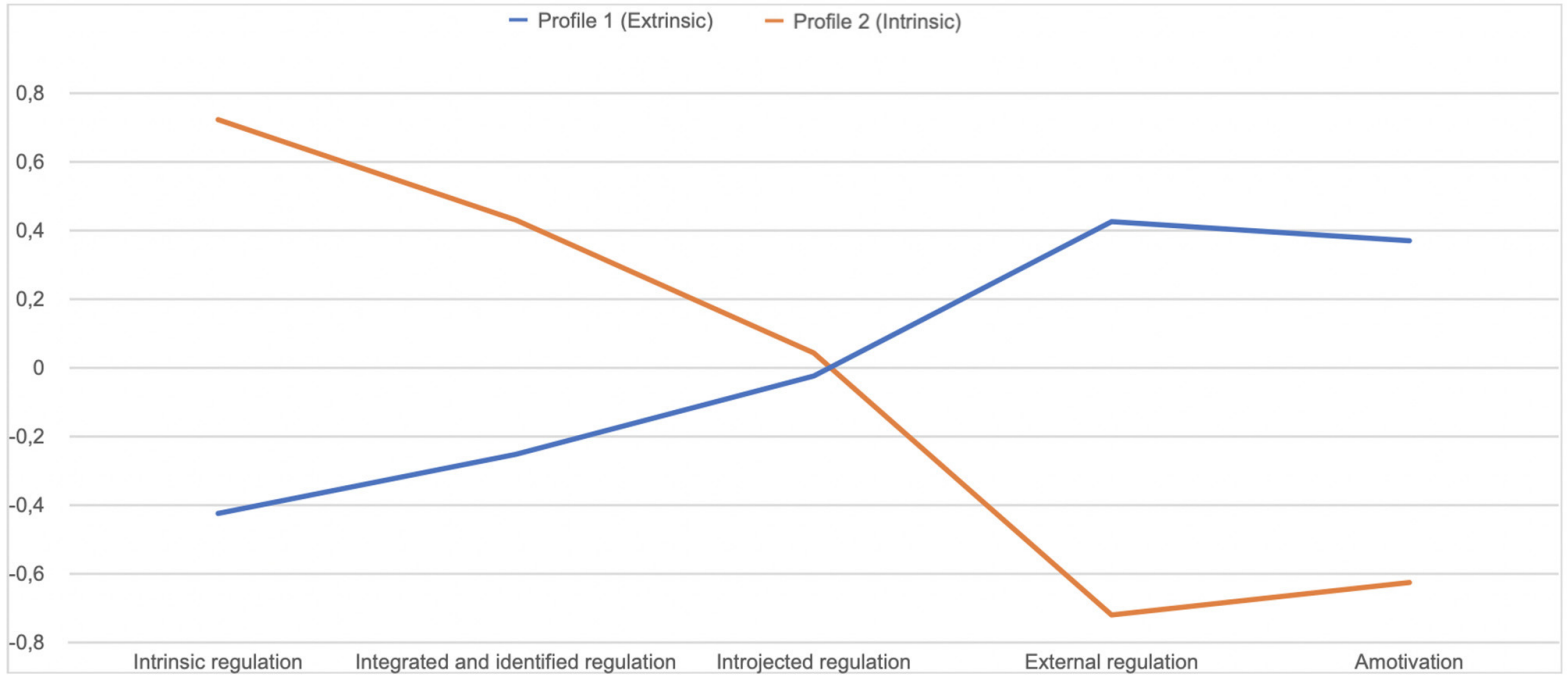
Profiles of regulatory styles (Study 2).

In the next step, latent profile analysis (LPA) (Rosenberg et al., 2019) was executed in order to estimate distinct HBMS profiles and extract different subgroups of respondents differing in terms of their regulatory style. The HBMS dimensions served as the basis for latent class extraction. In order to determine the adequacy of the model fit, three criteria were used: AIC (*Aikake information criterion*), BIC (*Bayesian information criterion*) (Akaike, 1973; Schwarz, 1978) and the measure of Entropy (Celeux and Soromenho, 1996). In case of AIC and BIC criteria, lower values indicate better model parameters and its higher predictive value (Akaike, 1973; Schwarz, 1978). In case of the measure of Entropy, values >.07 indicate acceptable classification accuracy (Jung and Wickrama, 2008). According to the values of the fit statistics (AIC = 2,944.81; BIC = 3,089.84; entropy = 0.81) the model with two extracted classes, presenting two distinctive profiles: “Extrinisic” and “Intrinsic” was with best fitted to data. Values of fit indices for all models tested are provided in Table 8. Figure 4 presents the mean values of the standardized variables in the extracted classes.

**Table 8:** Fit indices and entropy values for models tested in latent profile analysis, Study 2.

**Tested models**	**No of classes**	**AIC**	**BIC**	**Entropy**
Equal variances, covariances fixed to zero	2	3,357.84	3,414.44	0.88
	3	3,196.79	3,274.61	0.86
	4	3,155.06	3,254.11	0.83
	5	3,148.90	3,269.17	0.77
	6	3,140.19	3,281.68	0.79
Varying variances, varying covariances	2	2,944.81	3,089.84	0.81
	3	2,917.20	3,136.52	0.83
	6	2,833.28	3,275.44	0.90

In the first class the extracted “Extrinsic” profile was characterized by a lower level of intrinsic, integrated and identified regulation and a higher level of external regulation and amotivation. Second class, with “Intrinsic” profile, revealed higher level of intrinsic, integrated and identified regulation and lower level of external regulation and amotivation.

**Figure 5:**
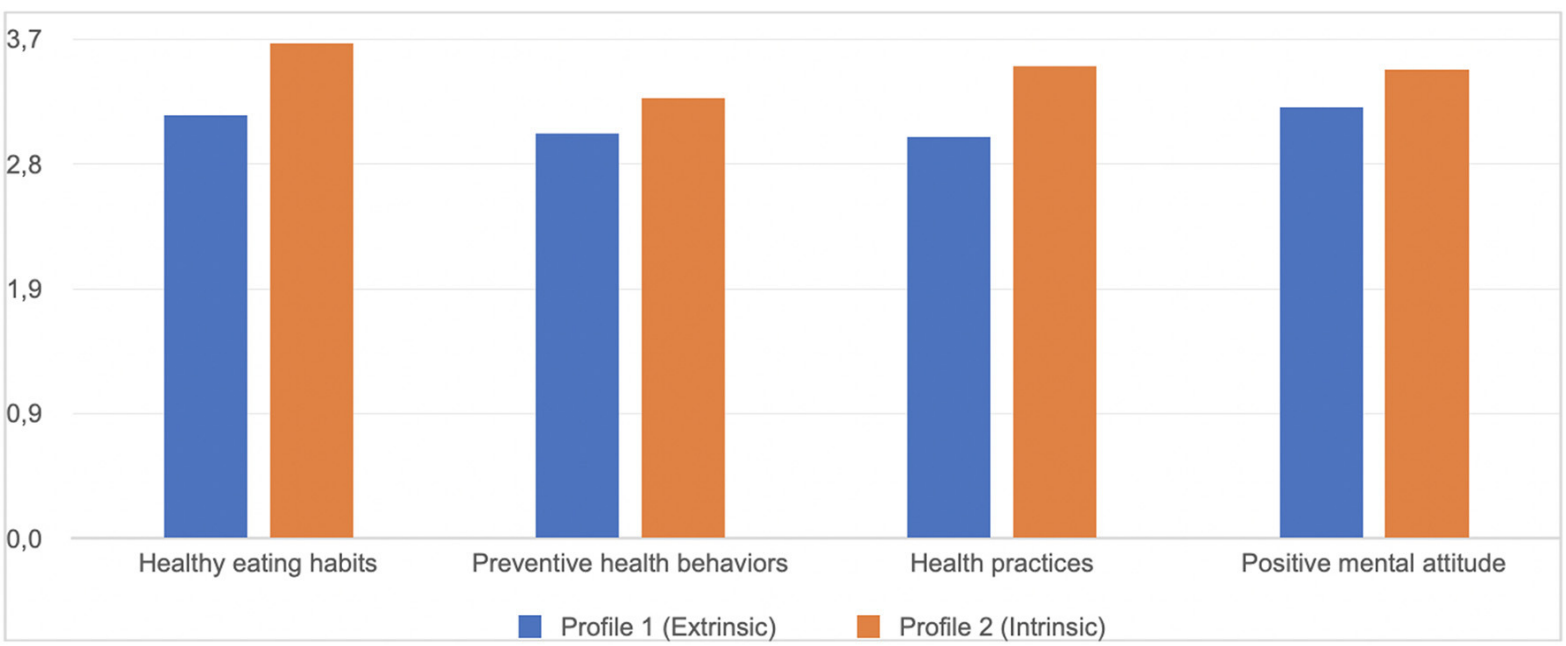
Mean values of health-related behaviors in the two extracted classes (Study 2).

Next, the two extracted classes were compared in terms of health-related behaviors (H7). The *t-*test for independent samples was performed. Both classes differed significantly, in terms of: healthy eating habits [*t*_(252)_ = −5.45; *p* < 0.01] preventive health behaviors [*t*_(252)_ = −2.53; *p* < 0.01], health practices [*t*_(252)_ = −4.56; *p* < 0.01] and positive mental attitude [*t*_(252)_ = −3.30; *p* < 0.01]. Mean values of each of the analyzed dimensions were significantly higher in the second class, with “Intrinsic” profile (Figure 5). The obtained results confirm H7.

### Discussion

The results of the validation procedure performed in Study 2 confirm the HBMS structural, convergent and discriminant validity.

The CFA analysis confirmed the model developed in EFA, proving its structural validity. All HBMS dimensions were characterized by a satisfactory level of reliability measured by Cronbach's α and ω coefficients. The matrix of relations obtained in the present study was similar to the one obtained by Ryan and Connell (1989). Regulatory styles theorized to be closer together along the motivational continuum (Ryan and Deci, 2000, 2017) were more highly correlated than those theorized to be distant. These results confirm H2. The strongest correlation coefficients occurred between the regulatory styles, that are more autonomous and associated with an internal locus of causality (Ryan and Deci, 2000). On the other hand, the high value of *r*-Pearson coefficient between introjected and external regulation may be related to the fact that both regulatory styles are associated with pressure: external (in the case of the external regulation) or internal (in the case of the introjected regulation) (Ryan and Deci, 2000). Because of these similarities, some researchers combine these two types of regulation into a more general controlled motivation composite (Williams et al., 1996).

Most of the expectations which concern the theoretical construct validity have been confirmed. Autonomous regulation of pro-health behavior is associated with setting intrinsic types of goals (especially health related) (Ryan and Deci, 2000) and proved to be stronger than other types of regulations associated with general motivation strength. Controlled regulation of pro-health behavior have generally weaker positive associations with intrinsic types of aspirations (Ryan and Deci, 2000) and with general strength of motivation. Amotivation was negatively related to intrinsic aspirations and promotional orientation and motivation strength, proving to be a good measure of lack of motivation. All the associations, however, were weak or moderate. This implies that the construct of regulation of health behavior is connected, but not interchangeable with both the concepts of aspirations and promotion and prevention self-regulation. These results confirm H3 and H4 (Kolańczyk et al., 2013). The relationships between the HBMS and the SATAQ-4 obtained in our study are also consistent with the assumptions of SDT theory (Deci and Ryan, 2000) and confirm H5 According to researchers (Ryan and Deci, 1985, 2000; Williams et al., 1996; Vallerand, 2000; Ryan et al., 2008) the SDT theory allows for multidimensional insight into the levels and types of motivation on hierarchically ordered different levels of regulatory styles. Thus, aside from the central division into external and internal motivation, it also captures aspects of separation into two general composites of motivation—autonomous and controlled. Within this division, the autonomous forms of regulation usually include intrinsic, identified and integrated regulation, whereas controlled forms of motivation include—introjected and external regulation (Williams et al., 1996). According to the researchers (Williams et al., 1996; Chrupała-Pniak and Grabowski, 2016) the main difference between external and introjected regulation is that in the former case, the pressure is external, whereas in the latter case, it comes from within. The results obtained in our study are not only consistent with SDT assumptions but are also in line with the results of other studies (Raposo et al., 2020) showing the relationships, between the regulatory styles and variables related to perceived pressure. Also, positive correlations between the different types of ideal appearance pressures exerted by family, media and peers with amotivation and introjected and external regulation confirm the theoretical validity of the HBMS.

The correlation coefficients between the HBMS sub-scales and the MBRSQ can also be considered a confirmation of the validity. The analysis of the obtained results allows us to conclude that a higher intensity of autonomous styles of regulation coexists with a more positive assessment of health and fitness and greater commitment to efforts to maintain good health and fitness. The opposite direction of dependence was obtained for external regulation and amotivation. Moreover, introjection was significantly associated with a higher degree of health and fitness orientation. This pattern of results was also obtained in the other studies, which analyzed the correlation between regulation styles and constructs such as positive and health outcomes (Williams et al., 1998; Levesque et al., 2007). An analogy in the results can be observed for all the analyzed dimensions of HBMS.

Finally, in the LPA, we observed two distinct profiles of participants. The first one—“Extrinsic”—was associated with a lower level of autonomous forms of regulation and a higher level of controlled forms of regulation. Second profile—“Intrinsic”—was the mirror image of the first profile and was associated with a higher level of autonomous forms of regulation and a lower level of controlled forms of regulation. The two classes differed in terms of health-related behaviors (H7). Participants with the “Intrinsic” profile were more health-oriented than participants with the “Extrinsic” profile. The results obtained in our study are in line with the results of other researchers (e.g., Hardcastle et al., 2015), applying a *variable-centered approach*, which rely on analyzing relationships between single regulatory styles and variables describing health-related behaviors. For instance, some studies suggest that amotivation toward health-related behaviors is associated with an inability to identify the reasons for acting and poor adherence to health behaviors (Thøgersen-Ntoumani and Ntoumanis, 2006). Low health behavior maintenance is also associated with external regulation (Ryan and Deci, 2000). Intrinsic regulation is in turn significantly and positively associated with positive health outcomes (Levesque et al., 2007). However, both the clinical practice and research show that while implementing change of health behaviors the regulatory style is fluctuating. Individuals implementing health behavior changes (eg. changing their eating habits) report being alternately amotivated and externally regulated with a focus on achieving external goals (Poraj-Weder et al., 2021). According to Ryan and Deci (2000), the boundaries between the regulatory styles are not firmly defined. People present a specific motivational profile, not a specific type of regulation (Teixeira et al., 2012). Thus, applying the *person-centered perspective* to understand how participants presenting various regulatory profiles differ in terms of health-related behaviors is a novelty of the study and its strength.

## Study 3

The aim of Study 3 was to analyze test-retest reliability of the HBMS. Specifically, we tested if scores on the HBMS are relatively stable over time.

### Procedure and Participants

The sample in Study 3 comprised 60 healthy adults (Table 1). Similar to Study 1 and Study 2, Study 3 was conducted individually using the paper-and-pencil method, in the presence of a researcher.

### Measures

Participants completed the HBMS twice with a 2-week interval in between the test and retest.

### Statistical Analyses

The analyzes used in Study 3 are presented in Figure 2. Statistical analyses were performed using IBM SPSS Statistics 26 software (IBM Corporation, 2019).

### Results

The aim of Study 3 was the assessment of the test-retest reliability that lets to verify if scores on the HBMS are relatively stable over time. For estimating test-retest reliability intraclass correlation coefficient (*ICC*) was used. The stability of scores over time was assessed in the study conducted with a 2-week interval in between the test and retest. The results of the reliability assessment of the HBMS is presented in the Table 9.

**Table 9:** Results of the reliability assessments of the health behavior motivation scale (HBMS), Study 3.

**HBMS scales**	***ICC***
Intrinsic regulation	0.84
Identified and integrated regulation	0.67
Introjected regulation	0.72
External regulation	0.85
Amotivation	0.94

*ICC, intraclass correlation coefficient*.

The intraclass correlation coefficients were in the 0.67–0.94 range.

### Discussion

The results of the study 3 show that the test-retest reliability of the HBMS (as measured by interclass correlation coefficient) was satisfactory. The obtained values of the interclass correlation coefficients of all subscales of the HBMS did reach the recommended in the literature (Liljequist et al., 2019) threshold of 0.50. The *ICC* values were mostly high or very high and all were satisfying, which proves that the HBMS is a reliable measurement tool. Thus, the HBMS ensures the satisfactory stability of the measurement of various forms of motivational regulation to undertake pro-health behaviors at different time-points.

## General Discussion

The aim of our study was to construct and validate the *Health Behavior Motivation Scale* (HBMS). Basing the HBMS structure on the applicable recommendations regarding the procedure of constructing diagnostic tools (Magnusson, 1991; Zawadzki, 2006; Brzeziński, 2007; Hornowska, 2007) allowed for its careful development in the scope of the adopted construction strategy and conceptual theoretical foundations, as well as for its linguistic validation. In addition, the linguistic and content analysis (Hornowska, 2007), as well as formalized content accuracy analysis (Zawadzki, 2006), along with the procedure of competent judges led to the elimination of incorrectly formulated linguistically and inaccurate items. The linguistic validation was carried out while the tool was being constructed. This enabled the development of its test version, which underwent psychometric properties assessment in the three studies (Study 1–3).

The studies 1–3 allowed for the verification of the HBMS psychometric properties. We posed seven hypotheses, all of which were confirmed. Internal structure of the HBMS was verified by means of confirmatory and exploratory (Study 1), and confirmatory (Study 2) factor analysis. The calculations were performed on various trials which allowed for the development of a stable and theoretically valid, five-factorial measurement model, describing the regulatory styles included in the motivational continuum by Ryan and Deci (2000, 2017). The reliability of the HBMS dimensions was assessed by evaluating its internal consistency by means of Cronbach's α and McDonald's ω coefficients (Study 1-Study 3) and the test-retest reliability (Study 3). The analysis of these results allows to conclude that the HBMS is internally consistent and ensures the satisfactory stability of the measurement. The Study 2 allowed for the verification of the HBMS construct validity (discriminant and convergent).

### Limitations of the Study and Recommendations for Future Research

Despite the fact that we confirmed the factorial structure, reliability, and discriminant and convergent validity of the HBMS, and all the psychometric indicators are on, at least, satisfactory level, the study has its limitations.

Firstly, the factorial structure of the HBMS differs from the theoretical Organismic Integration Theory structure of regulatory types. In the structure of the HBMS the identified and integrated regulations were recreated as one factor. Although research confirms that identified and integrated forms of regulations are highly positively correlated and the correlation between these two types of regulation is the highest among all the regulation types (Ryan and Deci, 2017) and some authors operationalize them as one, generalized dimension of external autonomous regulation (Deci and Ryan, 2000, 2008; Levesque et al., 2007), they are still considered theoretically distinct. The use of the HBMS does not enable to differentiate between these two types of regulations. It would be therefore beneficial for future research on health behavior motivation to complement the HBMS with the subscales measuring both types of regulation.

It should be also emphasized that the HBMS is a tool that diagnoses the regulatory styles without taking into account the goal content (Ryan and Deci, 2000; Teixeira et al., 2012). In the light of SDT theory, the distinction between the content of goals and aspirations (like overall well-being, physical fitness physical attractiveness, etc…) and different regulatory reasons (to conform, to maintain self-esteem, to have fun) is important for a comprehensive motivation diagnosis (Ryan and Deci, 2000, 2017; Teixeira et al., 2012).

Another limitation of the presented method is that it focuses solely on pro-health behaviors (serving to maintain or restore health), disregarding anti-health behaviors (causing direct or distant health damage) (Heszen and Sek, 2012). Due to the fact that taking care of health is associated with both, engaging in health-related behaviors as well as avoiding anti-health behaviors, it seems justified to develop a second variant of the HBMS that measures the motivation toward avoiding anti-health behaviors.

Finally, the creation of the HBMS was highly reliant on questionnaires as a method of conducting research. Data based on self-report are affected self-presentation of participants and can also be influenced simply by a lack of knowledge in the areas that are being explored (McDonald, 2008). When designing further research, it is worth linking HBMS with specific behavioral measures related to healthful behavior.

### Conclusions, Theoretical, and Practical Applications of the HBMS

The results of the present study show that the HBMS is a valid and reliable tool and can be successfully used in the population of healthy adults to measure motivation regulation toward health-related behaviors.

This tool has potential many applications in the health psychology area. The HBMS questionnaire can be used for both scientific and practical purposes. It can be a reliable and accurate instrument used in research which combines motivation and health psychology. It can also be used as a practical tool for healthcare professionals (psychologists, doctors, dietitians, diet coaches, and nutrition trainers). A key advantage of the HMBS is that it offers insight into an individual's motivational processes. This makes it possible to explain the mechanisms underlying the successes and failures of changing health-related behavior. Understanding these mechanisms may be used in practical way, by for example, helping to develop effective clinical intervention procedures resulting in profound and lasting changes in patients' health behavior (Halvari and Halvari, 2006). This can significantly contribute to increasing the effectiveness of implementing pro-health measures. The HBMS can find particularly useful in assessing an individual's readiness to engage in health behavioral change and setting motivational goals. Because each person's motivational status for health behavioral change is different, it is crucial to comprehend the types of an individual's motivational regulation styles and deliver an intervention tailored to each individual. The HBMS can serve as useful instrument to guide this process and enhance motivation to health behavioral change.

## Data Availability Statement

The raw data supporting the conclusions of this article will be made available by the authors, without undue reservation.

## Ethics Statement

The studies involving human participants were reviewed and approved by The Ethical Committee of Maria Grzegorzewska University. The patients/participants provided their written informed consent to participate in this study.

## Author Contributions

The research was conducted by MP-W and AP. MP-W, AP, and MS performed the analysis and wrote the first draft of the manuscript. All authors contributed to manuscript revision, read and approved the submitted version, conception, and design of the study.

## Conflict of Interest

The authors declare that the research was conducted in the absence of any commercial or financial relationships that could be construed as a potential conflict of interest.

## Publisher's Note

All claims expressed in this article are solely those of the authors and do not necessarily represent those of their affiliated organizations, or those of the publisher, the editors and the reviewers. Any product that may be evaluated in this article, or claim that may be made by its manufacturer, is not guaranteed or endorsed by the publisher.
