# Supplementary material for: The Development and Validation of the Health Behavior Motivation Scale
Source: Front Psychol. 2021 Sep 3;12:706495. doi: 10.3389/fpsyg.2021.706495 (PMC8446656; doi:10.3389/fpsyg.2021.706495)
Supplement: Supplementary file 1 [file Data_Sheet_1.docx]

**SUPPLEMENTARY MATERIAL**

| **TABLE 1A**\| Factor loadings acquired in confirmatory factor analysis based on preliminary factor structure | | |
| --- | --- | --- |
| HBMS dimensions |  | *f* |
| Intrinsic regulation | item 59 | .81 |
|  | item 54 | .88 |
|  | item 49 | .87 |
|  | item 45 | .81 |
|  | item 40 | .81 |
|  | item 33 | .83 |
|  | item 27 | .85 |
|  | item 21 | .83 |
|  | item 14 | .81 |
|  | item 7 | .79 |
| Integrated regulation | item 55 | .83 |
|  | item 50 | .76 |
|  | item 41 | .78 |
|  | item 35 | .64 |
|  | item 28 | .78 |
|  | item 22 | .82 |
|  | item 15 | .81 |
|  | item 8 | .71 |
| Identified regulation | item 36 | .83 |
|  | item 29 | .81 |
|  | item 23 | .80 |
|  | item 16 | .79 |
|  | item 9 | .78 |
|  | item 3 | .70 |
| Introjected regulation | item 60 | .61 |
|  | item 56 | .61 |
|  | item 46 | .82 |
|  | item 42 | .86 |
|  | item 37 | .83 |
|  | item 30 | .80 |
|  | item 24 | .70 |
|  | item 17 | .51 |
|  | item 10 | .77 |
|  | item 4 | .53 |
| External regulation | item 61 | .81 |
|  | item 31 | .84 |
|  | item 25 | .85 |
|  | item 20 | .85 |
|  | item 18 | .80 |
|  | item 12 | .77 |
|  | item 11 | .57 |
|  | item 5 | .67 |
|  | item 58 | .83 |
| Amotivation | item 53 | .73 |
|  | item 48 | .82 |
|  | item 44 | .78 |
|  | item 39 | .83 |
|  | item 32 | .86 |
|  | item 26 | .86 |
|  | item 19 | .85 |
|  | item 13 | .83 |
|  | item 6 | .69 |

| **TABLE 2A** \| Gender differences in the HBMS dimensions | | | | | | | |
| --- | --- | --- | --- | --- | --- | --- | --- |
|  | Women (*N* = 177) | | Men (*N* = 158) | |  |  |  |
|  | *M* | *SD* | *M* | *SD* | *t* | *df* | *p* |
| Intrinsic regulation | 3.05 | 1.34 | 3.13 | 1.42 | -.55 | 333 | .582 |
| Integrated and identified regulation | 17.81 | 7.20 | 17.92 | 7.27 | -.15 | 336 | .885 |
| Introjected regulation | 17.96 | 7.71 | 15.98 | 8.35 | 2.27 | 338 | .024 |
| External regulation | 10.62 | 8.16 | 10.53 | 8.48 | .10 | 336 | .919 |
| Amotivation | 7.31 | 7.63 | 7.04 | 8.01 | .32 | 336 | .752 |

| **TABLE 3A** \| Age differences in the HBMS dimensions | | | | | | | |
| --- | --- | --- | --- | --- | --- | --- | --- |
|  | Young adults (*N* = 221) | | Middle adults (*N* = 113) | |  |  |  |
|  | *M* | *SD* | *M* | *SD* | *t* | *df* | *p* |
| Intrinsic regulation | 3.08 | 1.43 | 3.09 | 1.30 | -0.11 | 332 | .912 |
| Integrated and identified regulation | 17.50 | 7.61 | 18.52 | 6.38 | -1.29 | 265.728 | .197 |
| Introjected regulation | 17.20 | 8.17 | 16.59 | 7.87 | 0.66 | 337 | .511 |
| External regulation | 10.35 | 8.22 | 11.10 | 8.47 | -0.79 | 335 | .433 |
| Amotivation | 6.64 | 7.77 | 8.32 | 7.77 | -1.89 | 335 | .060 |

**TABLE 4A** | Differences based on the place of residence in the HBMS dimensions

|  | Units up to 500 thousand residents  (*N* = 136) | | Units over 500 thousand residents  (*N* = 199) | |  |  |  |
| --- | --- | --- | --- | --- | --- | --- | --- |
|  | *M* | *SD* | *M* | *SD* | *t* | *df* | *p* |
| Intrinsic regulation | 2.99 | 1.40 | 3.15 | 1.36 | -1.07 | 333 | .287 |
| Integrated and identified regulation | 17.43 | 7.49 | 18.16 | 7.03 | -0.91 | 336 | .365 |
| Introjected regulation | 16.57 | 7.95 | 17.33 | 8.16 | -0.85 | 338 | .395 |
| External regulation | 11.34 | 8.31 | 10.06 | 8.27 | 1.40 | 336 | .163 |
| Amotivation | 8.54 | 8.30 | 6.26 | 7.31 | 2.61 | 269,782 | .010 |

| **TABLE 5A** \| Differences between higher education and other kinds of education in the HBMS dimensions | | | | | | | |
| --- | --- | --- | --- | --- | --- | --- | --- |
|  | Lower education (*N* = 164) | | Higher education  (*N* = 171) | |  |  |  |
|  | *M* | *SD* | *M* | *SD* | *t* | *df* | *p* |
| Intrinsic regulation | 3.08 | 1.38 | 3.09 | 1.39 | -0.08 | 333 | .934 |
| Integrated and identified regulation | 17.46 | 7.23 | 18.25 | 7.21 | -1.00 | 336 | .317 |
| Introjected regulation | 17.16 | 8.23 | 16.89 | 7.94 | 0.31 | 338 | .757 |
| External regulation | 11.21 | 8.22 | 9.98 | 8.35 | 1.37 | 336 | .172 |
| Amotivation | 7.65 | 7.89 | 6.75 | 7.70 | 1.06 | 336 | .288 |
